# Risk factor and symptoms of burnout in physiotherapists in the canton of Bern

**DOI:** 10.1186/s40945-019-0072-5

**Published:** 2019-12-21

**Authors:** Slavko Rogan, Yanni Verhavert, Evert Zinzen, Fabienne Rey, Aline Scherer, Eefje Luijckx

**Affiliations:** 1 Department of Health, Discipline of Physiotherapy, Bern University of Applied Sciences, Bern, Switzerland; 2 Academy of integrative physiotherapy und training education, AfiPT, Grenzach-Wyhlen, Germany; 3 Faculty of Physical Education and Physiotherapy, Vrije Universiteit Brussel, Brussels, Belgium

**Keywords:** Burnout, Physiotherapy, Prevention, Health promotion

## Abstract

**Background:**

Several studies have shown that the risk of burnout is high for people working in health professions. Many physiotherapists have either suffered from burn out personally or have seen colleagues suffer from it. In Switzerland, there is a lack of evidence concerning the risk factors and symptoms for burnout among physiotherapist.

The aim of this study was to empirically identify risk factors and symptoms of burnout in physiotherapists working in the canton of Bern.

**Method:**

Based on interview guidelines, three semi-structured interviews with physiotherapists who experienced burn out themselves were conducted. The questions were divided into two main categories: risk factors and symptoms. For analysis, the interviews were transcribed and assigned to individual categories.

**Results:**

High personal expectations and the pressure that comes with working on patients with chronic complaints were the most common answers from the participants. In this study these factors can be considered as important risk factors. In particular, emotional exhaustion and depression seem to be relevant burnout symptoms which lead to a decrease in personal performance.

**Conclusion:**

In this study physiotherapists with burn out working in an acute care hospital tend to suffer from symptoms of emotional exhaustion and depersonalization. For physiotherapists, sensitization to symptoms and risk factors of burnout is essential in daily work as well as in education. The results of this study might be of interest for physiotherapist or physiotherapists students to prevent and sensitive them for burnout symptoms.

## Background

In health professions, especially in physiotherapy, burnout has been recognized as an occupational hazard [1]. Burnout can be defined as a syndrome of emotional exhaustion, depersonalization and a sense of low personal accomplishment that leads to decreased effectiveness at work [2]. At least once in their career, physiotherapists can be confronted with a burnout syndrome either through experiencing burnout symptoms themselves or through colleagues suffering from a burnout syndrome [3–5]. Reasons why physiotherapists can be affected are therapeutic relationships that physiotherapists develop with their patients because patients often require an ongoing and intense level of personal and emotional contact. Lack of support from management or employees during physiotherapeutic work can lead to little recognition in work which is a risk factor for burn out [6–12]. High workload and time pressure are also important factors which can lead to a burnout syndrome [6, 7, 9, 10]. Burnout symptoms can lead to a limited workload ability of physiotherapists causing questioning the choice of their career and a decline in performance and/or quality of their work [7, 8, 10, 13]. The consequences of burnout in health professions are potentially severe for caregivers, health institutions, health insurance and can result in medical errors, depression, and adverse effects on patient safety [14].

Various countries, such as Australia, Austria, Cyprus, Poland and the USA have launched research projects in which treatment of burnout on the basis of its determinants is investigated [3, 9–11, 13, 15]. In Germany and Switzerland, there are no evidence-based approaches and models of how to design treatments for people suffering from burnout.

The aim of this study was to identify risk factors and symptoms of burnout in physiotherapy. From their point of view, recognizable and describable symptoms of burnout can be detected, contributing for developing diagnostic criteria.

## Methods

### Study design

The current study used a qualitative design to close the burnout research shortfall by means of a problem-centred interview. A self-created questionnaire in German was used. The questions were formulated based on the core content of previously conducted research in literature [16]. The categorization of questions was based on the principles of descriptive systems according to Mayring [17]. This categorization served as a basis to summarize and interpret the answers [18]. The validity check was carried out using a checklist developed by Konrad [16]. A trial interview was performed and recorded before starting the study. The questions (in English for the case of this manuscript) can be found in Table 1.

**Table 1:** Main categories and subcategories

Main categories	subcategories	Queries
Symptoms	Emotional states: Failure, anger, frustration, stress	Have you noticed that certain emotional states have occurred frequently?
	Behaviours: cynicism	Have you noticed any changes in your behaviour?
	Core symptoms according to Maslach: Emotional fatigue, depersonalization, personal fulfilment	1. How did you feel when you were in contact with other people? 2. How did you meet your patient? 3. How did you feel about your competence and your success?
	Other: Indifference in the job, questioning the career choice, decrease in efficiency, separation of work and private life, isolation tendency	Can you describe other symptoms that you have noticed?
Risk factors	Personal factors: Role of the giver, accepting patients and patients being able to help, regardless of the ailment, emotional states (feelings of failure and frustration), role conflict and ambiguity	What factors of your personality do you consider to be risk factors?
	Environmental factors: High workload in the absence of goal achievement, time pressure, quantitative overload, work with chronically ill patients, lack of support from managers and employees, exclusion from interdisciplinary decision-making processes	What have you seen in your environmental factors that may have favoured a development of burnout?
	Work locus of control: External focus	Where did you see possible causes of success? Failures at your work?
	Coping strategies: Emotion-oriented coping (EOC)	Were there any strategies that you used to live up to your needs and those of your environment?

### Participants

The recruitment took place by contacting acute hospitals, rehabilitation clinics, medical practices, consultants, psychotherapists and physiotherapy practices via e-mail. Physiotherapists working in acute care hospital or rehabilitation hospital in canton Bern who had suffered a burnout symptom during their working life were eligible for this study. Burnout was diagnosed by a physician. An informed consent form was signed by both the respondents and the interviewers prior to the interviews. Due to the study design, a clarification with the ethics committee was not necessary.

### Interview process and evaluation

Two explorative interviews were conducted at the workplace and one at home. Each interview was followed by a tape recording (“Zoom H1 Handy Recorder”) and a postscript was written. The interview was conducted in Swiss German. The time schedule for each interview was 30 min. We asked the physiotherapists to answer the questions so precisely as possible to get an accurate insight of the risk factors and symptoms. Interviews were transcribed and analysed using qualitative content analysis [17]. For transcription the program “audiotranscription f4 (License from Physiobern) was used. The interviews were translated from Swiss German into High German and transcribed without removing grammatical incorrectness. Furthermore, discourse particles such as “um”, “mhm” etc. were included in the transcription. The sections of the transcription were subsequently encoded and assigned to the created categories, and additional subcategories were formed in the event of a new name. Multiple answers were treated as important risk factors and symptoms of burnout.

## Results

### Participant

In total, 57 e-mails were sent. There was a response rate of 54% (*N* = 31). Six of the physiotherapists experienced burnout und could be included in this study. Finally, three physiotherapists were willing to participate (Table 2). All participants were able to complete their interview and answered all the questions. In addition, of one case a spouse was present with the interviewee who also participated in some questions.

**Table 2:** Characteristics of the participants

	Participant 1	Participant 2	Participant 3
Sex	Male	Female	Female
Age	43	57	36
Workplace	Acute care hospital	Acute care hospital	Acute care hospital
Work experience	18 years	25 years	11 years
Age at burnout	43 years old	57 years old	36 years old
Symptom onset	Mai 2012	2005	2011
Diagnosed with Burn out	February 2013	April 2005	Spring/Sommer 2011

### Interview results

In this study different symptoms could be observed. As for emotional state, emotional fatigue, emotional liability and need for withdrawal were answered in all three participants. A decrease in personal performance and depersonalization were also identified in all three participants. Furthermore, it could be demonstrated that two interviewed physiotherapists in this study tend to suffer more frequently from symptoms of insomnia, tension and concentration problems. As for risk factors we could observe that high self-expectation and perfectionism were important risk factors in all three participants. Work with chronically sick patients was identified in the subcategory environmental factors as a risk factor in two participants.

## Discussion

The aim of this work was to use a qualitative survey to describe the risk factors and symptoms of burnout in physiotherapists in the canton of Bern. Until today, numerous burnout models have been developed and defined [19]. Emotional exhaustion is described as the key criterion for a burnout diagnosis [1]. However, regarding burnout research it should be noted that it is difficult in a daily research routine to recruit persons with burnout syndrome for investigations. Because this topic can be associated with a considerable sense of shame, persons suffering from burnout do not like to talk about it [19]. The current study included three physical therapists with burnout syndrome from the canton of Bern. The symptoms and risk factors found in this study by physiotherapists, were based on the categories according Mayring [17]. Previous research on burn out could also identify these symptoms and risk factors. This shows the importance of these results for physiotherapists in Switzerland [19].

For health promotion and prevention, it is important to know essential determinants of burnout symptoms. However, it should be noted that potential determinants indicate a potential health hazard but do not necessarily lead to disease. Rösing [19] defined burnout as a self-perpetuating process. Burnout can occur when the workload becomes too high and the load capacity decreases. Maslach and Leiter [20] indicate that strong institutional characteristics (reward, community, fairness, values) must be present in the environment, so that burnout cannot occur. In the differential psychology individual approach, the aspect of personality is considered, and environmental factors (time pressure, lack of support) are hidden Schmidbauer [21] coined the term “helper syndrome”. The high self-expectations the physiotherapists in this study mentioned emphasized this aspect of Schmidbauer. On the one hand high expectations are made by the patient and on the other hand by the physiotherapists themselves. The motivation to help is especially noticeable in the field of physiotherapy. Physiotherapists are always required to provide optimal solutions to the problems of their patients. The risk here is that unrealizable goals are formulated which can trigger emotional overload peaks during the treatment process [10, 22, 23]. Working with chronically ill patients may be considered as another determinant [5, 22, 24]. This group of patients may cause false prognosis in physiotherapists. Due to a more personal relationship to patients with chronic diseases there is a potential higher risk of expectations from the patients on physiotherapists to be a problem solver [25].

The burnout symptom can be seen as a long-term stress episode. There is a close connection between stress and burnout. McEwen [26] postulated for stress an unspecific reaction of the body to any kind of request that the organism receives. Stressors show two different properties: a positive beneficial eustress and a negative harmful distress. If the distress persists for a longer period then symptoms such as emotional exhaustion, increased need for withdrawal, emotional liability, and a decrease in personal performance can occur [26]. The symptoms of the present studies can be found in the burnout models of Edelwich and Brodsky [25], of Lauderdale [26] and of the Maslach Burnout Inventory model [27]. As the triggering factor, stress can be considered. Age can be defined as the strongest variable that correlates negatively with burnout. The older the physiotherapists are, the less likely they are to be affected by the burnout symptom. In the current study, the physiotherapists suffering from burnout were more likely to be assigned to the younger and middle generation (Table 2). Persons over 40 years of age showed a significantly higher personal fulfilment [28]. In a study on older nurses, it was shown that the older generations learned to deal with issues encountered at the onset of careers and adapted to existing professional reality [29]. It was seen in our study that the physiotherapists had high expectations on themselves which caused emotional exhaustion. Maybe older physiotherapists have learned to deal with issues encountering their professional reality. The literature postulates that working conditions and coping opportunities of individuals should be optimized for the prevention of burnout symptoms [28, 30, 31]. Regular exchanges in the team or between employees can promote and develop sensitivity to burnout and maybe even prevent burnout syndromes. Sensitivity can apply to both physiotherapists and to the environment they work in. This includes the problem sensitivity and social sensitivity. Problem sensitivity is the ability to recognize problems in the foreground, to endure them, face them, and finally to develop problem-solving strategies. Social sensitivity is about interacting with other people. Strengthening these two sensitivities can consolidate a person’s position in an institutional setting.

A limitation of the study is the non-transferability of its results to the wider field of physiotherapy, since only three interviews with physiotherapists could be performed. These preliminary results should be incorporated in further, upcoming studies. More physiotherapist from Switzerland should than be included. Another limitation was that during one of the interviews the spouse was also present and answered some questions. This could have had an impact on the conversation content through the spouse’s verbal interjections, as well as non-verbal reactions which may have influenced the statements of the interviewee. According to Shenton [32], it is very important in qualitative studies that the framework conditions are defined at the beginning. All involved persons must be clearly defined, and the number of participants must be documented in the same interview.

## Conclusion

This study was the first qualitative study on burn out by physiotherapists in Bern. In this study the participants working in an acute care hospital tend to suffer more frequently from symptoms of emotional exhaustion and depersonalization. Over-high self-expectations and perfectionism, as well as working with chronically ill patients are identified as determinants of burnout in the investigated physiotherapists. The results of this study might be of interest for physiotherapist or physiotherapists students to prevent and sensitive them for burnout symptoms.

## Data Availability

The dataset used in this current study is available from the corresponding author on reasonable request.
